# Methods for Testing the Quality Attributes of Plant-Based Foods: Meat- and Processed-Meat Analogs

**DOI:** 10.3390/foods10020260

**Published:** 2021-01-27

**Authors:** David Julian McClements, Jochen Weiss, Amanda J. Kinchla, Alissa A. Nolden, Lutz Grossmann

**Affiliations:** 1 Department of Food Science, University of Massachusetts, Amherst, MA 01003, USA; mcclemen@umass.edu (D.J.M.); amanda.kinchla@foodsci.umass.edu (A.J.K.); anolden@umass.edu (A.A.N.); 2 Department of Food Material Science, University of Hohenheim, Garbenstrasse 21/25, 70599 Stuttgart, Germany

**Keywords:** vegan meat, protein, fiber, sustainability, anisotropy, laos

## Abstract

The modern food system is seeing a change in consumption patterns provoked by several drivers—including ethical, health, and environmental concerns—that are increasing the sales of meat analog foods. This change is accompanied by increased research and development activities in the area of plant-based meats. The aim of the present review is to describe methods that are being employed by scientists to analyze and characterize the properties of meat alternatives and to propose standardized methods that could be utilized in the future. In particular, methods to determine the proximate composition, microstructure, appearance, textural properties, water-holding properties, cooking resilience, and sensory attributes, of plant-based meat are given. The principles behind these methods are presented, their utility is critically assessed, and practical examples will be discussed. This article will help to guide further studies and to choose appropriate methods to assess raw materials, processes, products, and consumption behavior of meat analogs.

## 1. Introduction

There has been a tremendous increase in new animal-analog food products introduced into the food market recently, such as plant-based meat, fish, milk, eggs, and cheese. According to the Good Food Institute, a nonprofit organization, total retail sales of plant-based foods increased to $5 billion in 2019 within the U.S., which represents a 29% increase over two years [1]. Moreover, retail stores were reported to stock over 100 different meat analog products on their shelves. This increase in plant-based food products has largely been fueled by environmental sustainability, health, and animal-welfare considerations. For example, a study showed that diets high in meat and meat products produced more than two-fold higher carbon dioxide equivalent emissions (7.19 kg, CO_2_ e/day) than a vegan diet (2.89 kg, CO_2_ e/day) [2]. Furthermore, the additional health costs associated with high consumption of red and processed meat have been estimated to be around $285 billion in the U.S. alone [3]. However, many consumer perception studies in Europe have shown that the majority of consumers are currently unaware of the environmental impact of meat production, which shows that other drivers must also be playing an important role in the observed change in food consumption behavior [4].

The increased interest in food products that do not contain animal-based ingredients has resulted in a growth in research and development activity in the area of alternative food products, such as meat analogs. In the class of meat analogs, recent research activity revealed new raw materials, novel structuring techniques, and new product formulations [5,6,7]. However, there is still a lack of standardized methods to analyze, evaluate, and compare the new structures and foods that have been created. This may prevent efficient collaboration, adoption of results, and comparison with conventional foods, and thus slow down the development of new and improved meat analog foods. Therefore, this review aims to summarize and critically evaluate analytical methods that have been used by scientists to measure the composition, physicochemical properties, textural attributes, and sensory performance of meat analogs. It should be noted that many of these methods were developed by meat scientists, but lend themselves to adoption by researchers working on meat analogs. The review begins with a definition of meat and processed meat analogs. It then describes the usefulness of the different analytical methods available to characterize the properties of meat analogs, as well as the basic principles behind them, provides practical considerations and examples that could help promote their adoption. The review also lists relevant Association of Official Agricultural Chemists International (AOAC) methods that have initially been established for meats, but could be transferred to meat analogs with only minimal adjustments.

## 2. Definitions

In the meat area, two types of food classes are typically distinguished: fresh meat, meat preparations, and processed meat products. Fresh meat is the edible tissue of animals used for foods, whereas meat preparations are fresh meat that has been reduced to fragments with muscle fibers still present, and processed meat products are products that have been treated to increase the shelf life, convenience, diversity, and quality of meat without the typical appearance of fresh meat [8,9,10]. A similar classification will be adopted for the purpose of this review: meat analogs and processed meat analogs. In this sense, meat analogs and processed meat analogs will be defined as:

- *Meat analogs* are assembled from proteins, fats, carbohydrates, and other substances obtained from non-animal sources that are physically, enzymatically, or biologically structured to mimic whole muscle tissue, such as pork chops, chicken fillets, or beefsteaks.
- *Meat preparation analogs* are assembled from proteins, fats, carbohydrates, and other substances obtained from non-animal sources that are physically, enzymatically, or biologically structured to mimic fragmented whole muscle tissue, such as minced meat.
- *Processed meat analogs* are assembled from proteins, fats, carbohydrates, and other substances obtained from non-animal sources that are physically, enzymatically, or biologically structured to mimic processed meat products, such as sausages.

A variety of non-animal protein-rich sources may be used to assemble these products including plants, algae, fungi, or cultured meat. We should note that up to date it is still under debate whether cultured meat will be classified as meat or meat analog [9]. For the sake of convenience and brevity, we will summarize both meat analogs, meat preparation analogs, and processed meat analogs as “meat analogs” in this review.

## 3. Proximate Composition and Protein Characterization

Meat analogs are mainly composed of protein, fat, carbohydrates, minerals, and some minor compounds such as fibers, vitamins, and phytochemicals, but proteins play a critical role in determining their physicochemical and sensory attributes. Typically, the protein content of commercial meat analogs ranges from around 10 to 25% [11]. The development of alternative meat products requires the screening and selection of raw materials that are suitable for generating the desired structure (often anisotropy). Soy, pea, and gluten proteins are typically used for industrial analog production at present, but new protein-rich raw materials are being investigated, such as microalgae proteins, fungi, other plant proteins, and engineered meat tissues [12,13,14,15].

Analytical techniques and measurement protocols for meat analogs are required for a number of reasons. First, the selection of raw materials requires an appropriate type and amount of proteins to facilitate the formation of a meat-like structure, whereas carbohydrates may support structure formation, especially in extrusion and shear-cell processes [16,17]. Second, the final product needs to be analyzed to ensure correct labeling, depending on the local food and nutrition regulation requirements. Typically, the total protein, fat, dry mass, and mineral (ash) content are measured, and then the total carbohydrate content is determined as the remainder. However, meat analogs may also contain significant amounts of fibers from the plant materials used or because they are added to foster structure formation (see later). Third, protein characterization by sodium dodecyl sulfate-polyacrylamide gel electrophoresis (SDS PAGE), hydrophobicity assays, size exclusion chromatography, and other methods, enhances the understanding of the protein profile and properties that are necessary for the structuring process.

### 3.1. Proteins: Dumas- and Kjeldahl-Method, Composition, and Amino Acid Analysis

#### 3.1.1. Principles

Several protein assays have been established during the past one and a half centuries. They can be distinguished between the direct measurement of the amino acid content of a sample, and the indirect determination of proteinaceous nitrogen or spectrophotometric assays [18]. At the moment, spectrophotometric assays are not commonly used to analyze the protein content of meat analogs, because they require calibration with an appropriate reference material and are susceptible to interfering compounds [19]. In particular, the measured absorbance depends on the amino acid sequence of a protein, which depends strongly on protein type. Thus, these methods are not particularly suitable for the determination of the total protein content of meat analogs but are excellent methods for assessing a relative change in protein concentration, for example in solubility assays [20]. Commonly, most laboratories use the Dumas (e.g., AOAC 992.15) or Kjeldahl (e.g., AOAC 981.10 or 928.08) methods to determine the total protein content of the raw materials and final meat analogs:

- *The Dumas method* is based on the combustion of the food sample at temperatures between 900 and 1300 °C in an oxygen-rich atmosphere [21]. The released combustion gases (O_2_, CO_2_, H_2_O, N_2_, and NO_x_) are passed through gas- and water-sensitive traps/membranes to remove non-nitrogen containing gases. The nitrogen-containing gases are subsequently passed through a copper filled tube, which facilitates the reduction to N_2_. The remaining gas mixture is transported through a thermal-conductivity detector that delivers an electrical signal proportional to the nitrogen content.
- *The Kjeldahl method* is based on three steps: digestion, distillation, and titration. First, the sample is digested using boiling sulfuric acid and a catalyst, converting the nitrogen in the samples into ammonium ions. Second, the liquid is neutralized with NaOH, which promotes the release of ammonia gas in the subsequent distillation step by the reaction of ammonium ions with hydroxide ions. The ammonia is recovered by condensation and typically reconverted to non-volatile ammonium by a weak acid, such as boric acid. Third, the amount of recovered ammonium in the borate is determined by acid titration [22].

For the calculation of the protein content from the nitrogen content, a nitrogen-to-protein conversion factor is needed. The factor is established based on an *amino acid content analysis* (see practical considerations) [23]. Several methods for amino acid analysis are available but most commonly an acid hydrolysis (6 M HCl) in an oxygen-free environment is performed and the amino acids are separated in an ion-exchange HPLC system and detected with a ninhydrin post-column derivation [18]. Using this method, most amino acids are quantitatively determined, except for tryptophan, cysteine, and methionine. Tryptophan is easily degraded during acid hydrolysis and is therefore analyzed using alkaline hydrolysis before injection. Cysteine and methionine need to be oxidized into acid-stable methionine sulfoxide and methionine sulfone by performic acid prior to acid hydrolysis to prevent extensive acid degradation [24].

Finally, *SDS-PAGE* is carried out to characterize the protein profile (i.e., the different types and amounts of different protein fractions present) of the raw material and to detect possible changes in the protein composition during processing [25,26]. This is especially relevant for extrusion processes that impose harsh conditions, such as high temperature and shear stress, on the proteins’ structure and may promote crosslinking or degradation of the protein, which can be visualized by electrophoresis [27]. The proteins are separated on an acrylamide gel with a concentration between 8 and 12%. Many studies have used the conventional Laemmli sample buffer containing SDS and ß-mercaptoethanol with urea to solubilize the proteins and cleave disulfide bonds [25,26,27,28]. After electrophoresis, the gels are commonly stained with Coomassie brilliant blue to visualize the proteins.

#### 3.1.2. Practical Considerations

In this part, practical considerations will be given that will ease the adoption of these methods and enhance their reliability.

First, we will compare the *Kjeldahl* vs. the *Dumas* method. Both these methods are the basis for many reference methods published by associations, such as Cereals & Grains Association (AACC), AOAC, American Oil Chemists’ Society (AOCS), and International Organization for Standardization (ISO) [19] and have also been used to analyze the protein content in meat analog studies, especially to determine the protein content in the raw ingredients [29,30]. Traditionally, the Kjeldahl method has been used as a reference method, but the Dumas method has become established as a reliable alternative. The Dumas method has several advantages over the Kjeldahl method, including a higher throughput (analysis time usually 3–4 min vs. several hours), safety concerns (no handling of caustic acids, no toxic chemicals), and lower costs per sample [19]. However, the Dumas instruments available on the market also require regular maintenance and exchange of consumables, such as copper and adsorbers, which requires trained staff.

Both methods can be used to analyze different sample sizes and nitrogen (protein) concentrations and both need calibration with a standard (ethylenediaminetetraacetic acid (EDTA) or tris(hydroxymethyl)aminomethane (THAM) for Dumas, pH calibration/indicator for Kjeldahl). Dumas instruments can detect between 0.003 and 50 mg nitrogen, and fit sample sizes between a few mg up to 2–3 g, whereas Kjeldahl has a lower limit of quantification of about 0.02 mg nitrogen [31]. Since sample amounts are quite low, a homogenization step (using e.g., a small blender) prior to the measurement is essential for meat analogs to ensure a homogenous sampling for both methods.

The choice of a correct nitrogen-to-protein conversion factor is a critical step in calculating the total protein content from the nitrogen content for both methods. While both methods also detect non-proteinaceous nitrogen, Kjeldahl only detects organic nitrogen compounds, NH_3_, and NH_4_^+^, but Dumas determines all nitrogen-containing components [32]. For most plant proteins, factors are commonly reported to be between N × 5–6 [30,33]. However, nitrogen-to-protein conversion factors have not been published for many new raw materials, which may result in the reporting of erroneous protein contents.

For new raw materials, the conversion factor can be established based on the amino acid composition. Two factors are commonly used *k*_p_ and *k*_a_: here, *k*_p_ is the ratio of the sum of the weights of anhydrous amino acid residues to total nitrogen content (protein nitrogen + non-protein nitrogen), whereas *k*_a_ is the ratio of the sum of anhydrous amino acid residue weights to the sum of only the nitrogen found in the detected amino acids [34]. Each of the two factors has its limits: *k*_p_ may underreport the actual protein content because it depends on the recovery of amino acids during the analysis, while *k*_a_ may overestimate the actual protein content because it does not take into account the non-protein nitrogen. For these reasons, it is recommended to average both factors to obtain a mean *k* factor for nitrogen-to-protein conversion. Moreover, as prosthetic groups can also contribute to the total protein content, scientists may also take protein-associated groups into account [33]. In addition, protein quality indices, such as the protein digestibility-corrected amino acid score (PDCAAS) or digestible indispensable amino acid score (DIAAS), should be considered because they vary between the different new protein sources. The DIAAS value is recommended by the Food and Agriculture Organization of the United Nations (FAO) and is determined using the true ileal amino acid digestibility of indispensable amino acids in the food protein compared to a reference protein [35]. For example, the DIAAS of soy protein is 84, whereas wheat protein has a lower score of 45 [35]. Such scores can influence the labeling of protein values in specific countries, such as the packaging statement “good source of protein” [36].

Second, when analyzing the *amino acid content*, a few things should be considered even though amino acid analysis is an established method in many laboratories. Some amino acids are prone to degradation during acid hydrolysis, especially cysteine and methionine. For quantification, cysteine and methionine need to be oxidized prior to acid hydrolysis, which enhances their chemical resistance [37]. Tyrosine is prone to acid degradation, which can be minimized by the addition of phenol [38] and asparagine and glutamine are deamidated during hydrolysis resulting in only one accumulated value for both asparagine/aspartic acid and glutamine/glutamic acid residues [24]. Additionally, to distinguish between proteins and free amino acids, the proteins are separated from the amino acids by employing a selective precipitation step using a polar solvent (e.g., ethanol or acetone) or trichloroacetic acid, and the amino acid content in the supernatant is analyzed [39].

Last, for *SDS PAGE*, the Laemmli buffer is useful to maximize the solubility of proteins and to compensate for differences in their charge and conformations, but it may not represent the real aggregation conditions of proteins since aggregates are re-solubilized by SDS and ß-mercaptoethanol. Using non-reducing (i.e., without β-mercaptoethanol) or even native conditions should therefore be considered for some studies. Moreover, if the protein concentration in single bands is too low, proteins are not sufficiently dyed by Coomassie blue. In this case, other staining techniques, such as a silver staining, should be considered, which has a 10-fold increased sensitivity.

### 3.2. Total Fat and Dry Matter Content

#### 3.2.1. Principles

Fats contribute to the nutritional, structural, and flavor profile of the analog, but are still difficult to replace because it is hard to mimic the structured fat from animals [40]. Processed meat analog foods typically contain up to 20% fat, which is commonly analyzed using a conventional Soxhlet extraction after determination of the dry matter content [11,41]. The most straightforward method to measure the dry matter content is to grate the sample with a defined amount of sea sand and subject both to oven drying at >100 °C overnight or longer (e.g., AOAC 950.46). After letting the sample cool down in a desiccator, the dry matter is measured by differential weighing.

To determine the total fat content in the dry matter, a *Soxhlet extraction* method is employed. Samples are placed in an organic thimble and soaked automatically for several cycles in a nonpolar solvent, such as hexane or petroleum ether with a low boiling point, which solubilizes the fat in the solvent (e.g., AOAC 960.39). The solvent is removed from the sample by an overflow, releasing the fat into the solvent container. After complete extraction, the solvent is removed from the device by distillation and may be reused for another analysis cycle. The extracted fat is subsequently determined gravimetrically. To decrease the extraction time, devices are available that utilize sonication to breakdown the structure, or apply high-pressure to achieve higher boiling points and consequently higher extraction temperatures [42].

If the fat is bound to a protein matrix and is not fully extracted by the solvent, then a simple Soxhlet extraction will result in underreporting of the total fat content (e.g., AOAC 922.06). To release the bound fat, a hydrolysis with boiling 4 M HCl before the Soxhlet extraction will yield the total fat content, which is often referred to as the *Weibull-Stoldt method* [43]. In this method, samples are hydrolyzed in a manual reflux heater equipped with an air cooler or an automatized hydrolyzer, the excess acid is washed out, and then the samples are dried before the solvent extraction step.

#### 3.2.2. Practical Considerations

An important requirement for extracting lipids from meat analog materials is the removal of water before extraction, which if not removed would minimize the solvent-sample contact area. The easiest way to remove the water is by oven-drying. In practice, homogenized samples (i.e., with a pestle and silica sand) are transferred to the Soxhlet thimble after they have been dried overnight or longer at above 100 °C to determine the dry matter content. If samples were acid hydrolyzed prior to the Soxhlet extraction, the filters need to be dried completely before the extraction procedure [44].

In case the sample has a low-fat content (<0.1%), the exact fat content is difficult to measure, since it is a gravimetric method. Most analytical balances have a readability of around 0.1 mg, which typically results in reliable results for sample weights before the solvent extraction of around 10 g. In the case of very low-fat contents, the sample size may be increased depending on the maximum thimble size available for the Soxhlet extractor.

### 3.3. Ash Content (Total Minerals)

#### 3.3.1. Principles

The ash content of meat analogs, which is sometimes also referred to as the total mineral content, is an important parameter that influences their nutritional and quality attributes. These minerals may come from the raw materials (such as protein concentrates/isolates) or they may be added as a seasoning (such as salts). The total amount of minerals is determined gravimetrically after the incineration of the sample which combusts all the organic compounds, leaving only the ash (e.g., AOAC 920.153). Typically, a wet solid sample is first homogenized with magnesium acetate in dried porcelain dishes to loosen the material and then dried to remove residual water. Subsequently, the samples are incinerated at a high temperature to remove any organic compounds from the non-volatile minerals. The whole process usually takes several hours, and the final incineration is carried out overnight or longer to ensure complete combustion. After complete combustion, the ash is gravimetrically measured with similar limitations as given above for the total fat content determination [45].

#### 3.3.2. Practical Considerations

Even though the method is relatively straightforward, a number of things should be carefully considered to obtain accurate results. First, the complete removal of all organic compounds is essential for an accurate determination of the total mineral content. The final combustion should be carried out long enough, and the time needed to fully incinerate may vary from sample to sample. Usually, samples are combusted for several days but initially, the minimum time to reach weight equilibrium (i.e., the sample is fully incinerated) can be analyzed to minimize the time of the analysis. Second, the sample dishes need to be dried in an oven and cool downed in a desiccator prior to analysis to remove any residual water that would falsify the blank value of the dish before sample loading.

As a side note, it should be noted that, for some applications, specific minerals need to be analyzed, for example, the sodium or potassium content as a taste agent. This is usually done using atomic emission spectroscopy or ion chromatography.

### 3.4. Minor Compounds

Meat analogs also contain other minor compounds, such as fibers, minerals, and vitamins. The total fiber amount can be analyzed using methods such as AOAC 985.29, AOAC 2009.01, AOAC 2017.06. These methods are typically based on simulating the human digestion until the large intestine. They first simulate the breakdown of dried and defatted food material by enzymes (amylases, proteases, amyloglucosidases) followed by a precipitation and filtration of the undigested material that yields the high molecular weight dietary fiber after protein and ash determination. The low molecular weight soluble dietary fiber that remains in the permeate can be analyzed by HPLC [46]. To separate the soluble (high and low molecular weight) from the insoluble fiber, a filtration step is added before ethanol precipitation.

The diverse set of minerals and vitamins present in meat analogs are analyzed by various kinds of different methods (spectroscopic, chromatographic, atomic emission spectroscopy, etc.) and the interested reader is referred to textbooks for more information [47,48].

### 3.5. Infrared Spectroscopy for Fast Proximate Composition Analysis

Near-infrared (NIR) spectroscopy has been successfully used to analyze the main constituents (e.g., water, fat, and protein) of meat and meat samples for a few decades [49,50]. The successful implementation in many studies was followed by the release of an official AOAC method using the FOSS FoodScan™ (AOAC 2007.04) device that employs near-infrared transmission measurements coupled with the FOSS Artificial Neural Network Calibration Model [51]. The main advantages of the NIR technique are the rapid analysis time, its potential use as an in-line measurement technique, and its non-destructive nature, but it requires extensive calibration. However, research to date has not yet determined the feasibility of NIR measurement techniques for meat analogs. This knowledge gap should be filled to ensure the fast adoption of NIR in production processes for meat analogs.

### 3.6. Differential Scanning Calorimetry

#### 3.6.1. Principles

Differential scanning calorimetry (DSC) is used to detect phase transitions, such as protein denaturation or fat crystal melting [52]. The use of DSC methods is essential for the development of meat analog products for two reasons. First, the majority of proteins from alternative sources (i.e., from plants, insects, fungi, algae, single cells, or cellular agriculture) are globular. To create a fibrous structure, the proteins are denatured and re-aligned in a shear field [53]. As each protein has its unique denaturation temperature, knowledge about the phase transition of the protein is essential to design a process that yields a fibrous structure. Second, meat and processed meat does not only consist of protein but also plant-based oils and fats. One approach to mimic the structured fat tissue found in animals with its unique structure and melting behavior is to blend solid fat and liquid oils to achieve the desired mouthfeel and melting behavior. Another approach is to use structuring techniques, such as enzymatic modification of lipids or using oleogels [54]. To analyze the melting behavior of structured fats designed to be incorporated into meat analog products, DSC measurements are usually carried out [55].

**Figure 1:**
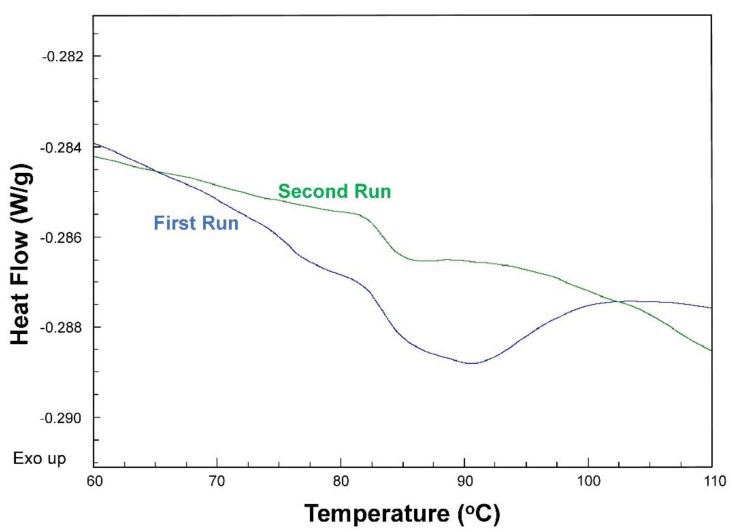
Differential scanning calorimetry (DSC) profile (heat flow versus temperature) of fava bean protein solutions when heated at 1 °C min^−1^. The solution was heated (first run), cooled, then heated again (second run), which shows the thermal transitions are partially irreversible.

The heat-flux DSC technique is based on assessing the change in $c_{p}=\frac{dq}{dT}$ (heat capacity) by heating the sample and a reference (usually water or air) simultaneously over a controlled temperature range. The lack of any phase transition in the reference results in a sudden change in the relative heat capacity as the sample undergoes a phase transition (Figure 1). Melting or denaturation is evident by the presence of latent heat, whereas glass transitions do occur without the presence of latent heat, and are thus characterized as a second-order transition with a sudden jump in the heat capacity [56]. The change in heat capacity is then used to analyze enthalpy, entropy, and Gibbs free energy changes [57].

#### 3.6.2. Practical Considerations

The unfolding of proteins is characterized in many cases by an endothermic and endergonic phase transition, meaning energy needs to be supplied to the system and it is a non-spontaneous process [58]. Most proteins are relatively fragile systems, which are stabilized by a subtle balance of covalent and non-covalent interactions that favor the native conformation (disulfide bonds, hydrogen bonds, hydrophobic interactions, van der Waals attractions, and electrostatic attraction between oppositely charged groups) and those that favor the denatured state (conformational entropy, hydration, bond bending/stretching, and electrostatic repulsion of similarly charged groups). It is estimated that the balance between these two energies ranges under physiological conditions is between −20 and −65 kJ mol^−1^, which is far less than the strength of one covalent bond (around −500 kJ mol^−1^) [59]. Thus, even small energy inputs may result in the unfolding of globular proteins. Often, common DSC devices are unable to detect these small energy changes. If possible, higher protein concentrations or larger sample pans that are suitable for higher sample amounts (for some devices up to 160 μL) should be considered, as well as identifying the optimum heating rate. However, for many proteins, the resolution is still too low and DSC microcalorimeters should be employed to detect protein denaturation in such systems.

Additionally, environmental conditions of the protein sample need to be considered for a reliable detection of the denaturation temperature. Factors such as pH, ionic strength, and heating rate are known to influence the denaturation temperature and should be kept at the same conditions as during the structuring process [60,61].

### 3.7. Practical Examples of Utilization of Proximate Analysis Methods

Most researchers who investigate the formation of fibrous meat analogs have utilized the Dumas and Kjeldahl method to analyze the protein content of the raw material used in the production process [26,29,62,63,64]. For established protein sources (such as pea or soy protein), nitrogen-to-protein conversion factors can simply be taken from published literature data, while for novel protein sources (e.g., microalgae and insects) these values may have to be measured, although some have been recently reported [23,65,66]. Some authors still use a conversion factor of 6.25, which initially assumed a nitrogen content in proteins of around 16%, which is inaccurate for most protein sources and may lead to over- or under-estimation of the true protein content (see above) [12,32,67].

In a recent work of Chiang et al. [29], the authors produced meat analogs by high moisture extrusion using soy and gluten protein and analyzed the protein as well as the dry matter content of the final meat analog at different soy and wheat gluten ratios. Even though the protein content remained almost constant at different soy/gluten ratios, the fiber formation was significantly influenced by the addition of gluten. Higher gluten content (20 or 30%) resulted in extensive fiber formation and interconnected fibers. Moreover, the moisture content was considerably lower compared to boiled chicken breast (69% and 57%, respectively), which may influence structure formation and sensory aspects due to the presence of carbohydrates or mineral residues [68].

In a study using soy proteins, it has been reported that protein–protein cross-linking begins in the extruder and not within the cooling die [27]. This conclusion was drawn by taking samples at different stages in the extrusion process and subsequently separating and visualizing the protein composition at different extrusion levels on an SDS-PAGE gel. The results showed that proteins were already significantly aggregated in zone 3 (of 5) at 150 °C at a moisture content of 29%. Conversely, proteins were less aggregated at moisture contents of 60%, showing the presence of different protein–protein interactions in high and low moisture protein analogs [69].

## 4. Texture and Sensory Properties

The unique textural properties of meat are caused by the complex scaffolding of the muscle tissue consisting of repeating structures based on sarcomeres that are eventually grouped into muscle fibers and surrounded by connective tissue, the cell membrane, and, depending on the meat type, stored intramuscular fat tissue [17,70]. Chefs and food scientists have tried to mimic these unique structures for centuries, but considerable texture advances have been made recently due to the introduction of novel extrusion and shearing technologies, as well as by the use of new raw materials [53]. The main challenge is to create the fibrous, meat-like structure from globular proteins and to structure the fat phase with plant-based oils and fats. Hence, scientists have used a variety of different techniques to assess the structural properties of meat analogs that will be reviewed below.

### 4.1. Rheometry: Rotational, Closed-Cavity, and Capillary Rheometers

Rheology is a powerful tool to understand how a material behaves under shear stress and strain. In some cases, rheological measurements can provide insights into meat analog processing because the material is exposed to shear and temperature gradients over certain times, which is especially relevant for extrusion and three-dimensional (3D) printing processes [71]. *Rotational rheometers* are typically classified as either controlled strain or controlled stress instruments. In a controlled strain rheometer, the sample is sheared by a lower plate using a defined strain or rotational speed, and the torque to hold the upper geometry in the same position is measured and transferred into shear stress. Conversely, a stress-controlled rheometer uses only the upper geometry to generate shear stress, while the lower one is fixed. The motor generates a defined torque that is converted into stress, while the strain or rotational speed is measured by the angular displacement of the geometry analyzed using a laser [72]. Additionally, closed-cavity rheometers have been used to study the deformation behavior of protein dispersions using oscillatory measurements. The advantage of the closed-cavity technique is that it is able to combine high temperatures and pressures while preventing moisture loss, which is similar to extrusion conditions, while also maintaining an even shear stress across the sample because of the bi-conical design [73,74].

Beyond rotational rheometers, food scientists have used *capillary rheometers*, which are especially suitable to study the flow behavior at higher shear rates (>10^2^ s^−1^), flow instabilities, and observe wall slip effects, and are thus relevant for extrusion processes [75,76,77,78]. Capillary rheometers consist of at least one piston, a barrel to load the sample, and a capillary die. Additionally, the device is equipped with pressure, displacement, temperature, and force sensors [76]. The apparent viscosity is calculated from the wall shear stress divided by the apparent wall shear rate, which is obtained by measuring the total pressure drop caused by the capillary and at the capillary entrance (which will yield shear stress) and the piston speed/volume flow (which will yield shear rate) [79]. To compensate for entrance and exit effects, wall slip, and a non-parabolic velocity profile, Bagley, Mooney, and Weissenberg–Rabinowitsch corrections need to be taken into account to obtain true viscosity values [80].

**Figure 2:**
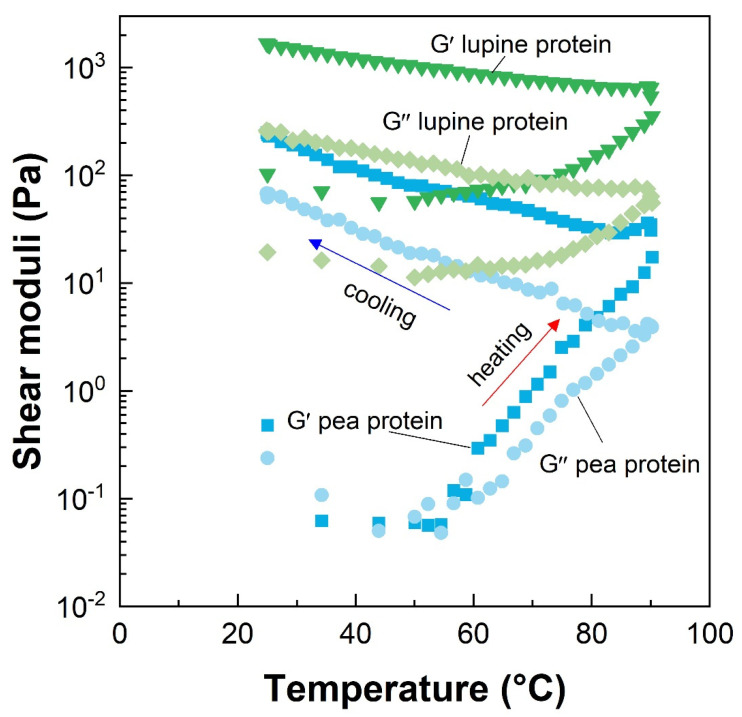
Temperature sweep in a parallel plate from 25 °C to 90 °C at a heating rate of 5 °C min^−1^ of pea (12.5 %) and lupine (15 %) protein dispersions at *γ* = 0.1% and *f* = 1 Hz.

Next, we describe common measurement techniques that are used to analyze the behavior of meat analog materials under flow and deformation:

- *Flow curve:* During a flow curve measurement, the samples are sheared under rotation with different measuring geometries such as a double gap or concentric cylinder systems, depending on the sample. The sample is sheared at increasing shear rate levels and the resulting shear stress is measured or vice versa. The obtained stress vs. rate profile is used to calculate the apparent shear viscosity and yield stress. As many food materials do not show Newtonian flow behavior (i.e., the viscosity depends on the shear rate or on the time of exposure at a constant shear rate), the flow curve provides valuable insights into the behavior of the material during shear conditions. These insights are valuable to understand the material behavior in an extruder and the specific mechanical energy input that influences the viscosity of the sample during extrusion [81,82]. However, as shear rates are limited during rotational rheometry, capillary rheometers are often better suited to obtain viscosity values that are present under extrusion conditions.
- *Amplitude sweeps:* Strain or amplitude sweeps are conducted in the oscillatory mode of a rheometer. The sample is positioned in the gap and the amplitude of the oscillating measuring geometry is increased at a constant frequency. Amplitude sweeps are useful to define the linear viscoelastic range (LVR), in which the material response (i.e., storage and loss modulus) remains constant and no breakdown of the material occurs. Beyond the linear viscoelastic range, the material starts to break down and starts to flow. The obtained curve can be used to calculate the yield point (limit of the linear region) and the flow point (crossover point at which storage modulus equals loss modulus). Thus, comparing the magnitude of the linear viscoelastic range between materials helps to understand under which conditions an irreversible breakdown of the structure occurs. Moreover, amplitude sweeps are useful to characterize the state of the material. A typical example is that of a gelled product, which is characterized by a higher storage modulus than loss modulus in the linear viscoelastic range. Additionally, amplitude tests have recently been used in meat analog research for the development of fat replacer systems [40,83].
- *Frequency sweeps:* Frequency sweeps are carried out in oscillatory mode using a parallel-plate or a cone-plate measuring geometry in the linear viscoelastic region (i.e., non-destructive conditions) to analyze time-dependent (relaxation) behavior and acquire insights into material properties. The method is based on shearing the sample at constant strain and increasing the angular frequency, typically from 0.1 to 100 s^−1^ [84]. This increase reveals the time-dependent behavior of the sample, since at low frequencies the material has more time to relax, while less time is available at higher frequencies. Commonly, the moduli of more solid-like materials (e.g., gels) are less frequency-dependent than more liquid-like materials (e.g., fluids or slurries). Thus, frequency sweeps can provide insights into relaxation times, the behavior of the sample under storage and processing conditions, the amount, and types of crosslinks that are present in meat analogs, and are useful to compare the effects of structuring techniques on the protein- and fat-phase [6,40].
- *Temperature sweeps:* During temperature sweeps, the sample is heated and/or cooled while being sheared under constant strain and frequency conditions in oscillatory mode (Figure 2). This method is employed to detect structural changes in the material during a heating or cooling step, such as the onset of gelation or melting behavior. To detect these changes, the measurements have to be carried out in the linear viscoelastic region to maintain the integrity of the sample. Additionally, samples need to be protected against moisture loss from evaporation by covering their exposed surfaces with silicon oil or by using a solvent trap that is available from most rheometer manufacturers. After placing the sample in the gap, the sample is heated by a Peltier element and, in some cases, a hood that applies convective heat is placed around the geometry [6]. Depending on the aim of the experiment, the sample may start in a liquid or solid form prior to analysis to detect any thermal events, such as aggregation and gelation. Both approaches give valuable insights into the processing of meat analogs. First, heating a liquid protein dispersion will result in denaturation resulting in a sol-gel transition at a specific temperature, which needs to be reached during extrusion or other structuring techniques to achieve protein crosslinking [27]. Second, re-heating a gelled material reveals the type of bonds present (e.g., hydrophobic, hydrogen bonds, or covalent) in the material that are responsible for structure formation [84].

- *Large amplitude oscillatory shear:* The above-described methods (frequency and temperature sweeps) are typically conducted in the linear viscoelastic range and are classified as small amplitude oscillatory shear (SAOS) methods. However, during the processing of meat analogs, especially during extrusion, the material is sheared beyond the linear region. To understand the behavior in the nonlinear region, large amplitude oscillatory shear (LAOS) experiments are used to ascertain the behavior of the material under high strains that mimic those real products may experience, e.g., shearing, extruding, cutting, or chewing [85]. The sample is sheared in oscillatory mode using a constant frequency (often 1 Hz) at increasing strain, commonly from 0.1 to 1000% [85,86]. The material response in LAOS experiments is not analyzed by evaluating the behavior of stress and strain over time (as in SAOS experiments), but the stress is plotted against the strain or strain rate, which yields so-called Lissajous plots. Detailed examples and interpretations of such plots can be found in Schreuders [86]. In general, the plots give insights into the viscous and elastic behavior of the material at different strains, which is helpful to understand the material characteristics for extrusion and shearing processes. For example, concentrated pea protein isolate dispersions had an elastic behavior at small strains (1.6%), while a more viscoelastic response was measured at intermediate strains (85.4%) and a plastic behavior at high strains (735.2%). Moreover, the dissipation ratio was calculated to display the ratio of elastic and viscous components during increasing strains, calculated from the area of the viscoelastic and perfect plastic response in the Lissajous plots. While low dissipation ratios indicate a mainly elastic behavior, higher dissipation ratios (>0.8) imply more viscous components present in the material [86].

### 4.2. Normal Force Tests: Tensile, Compression, and Cutting Tests

For many purposes, it is important to characterize the textural properties of meat analogs when a normal force is applied, i.e., a force that is perpendicular to the surface of the material being tested. This type of experiment is usually carried out using instruments known as texture analyzers. These machines are built with a movable load cell that measures the force needed to compress or elongate the test material. Typically, a stress vs. strain or time profile is acquired when the load cell is moved up and/or down. The physical material properties that can be obtained from these curves include hardness, tensile strength, adhesiveness, brittleness, gumminess, and more. For meat analog research, these parameters are important for two main reasons. First, by comparing compression and elongation forces of meat analog materials with regular meat products, material, and process conditions can be tuned to obtain the same textural properties as in regular meat products. This knowledge is important to create meat analogs that behave similar to meat products, e.g., have similar cutting or mouthfeel attributes (e.g., bite strengths). Second, elongation tests provide direct insights into the extent of anisotropic structures present in the meat analog (i.e., the response of the material depends on the direction that the force is applied). Thus, normal force tests should be a part of the toolbox of meat analog scientists and developers.

**Figure 3:**
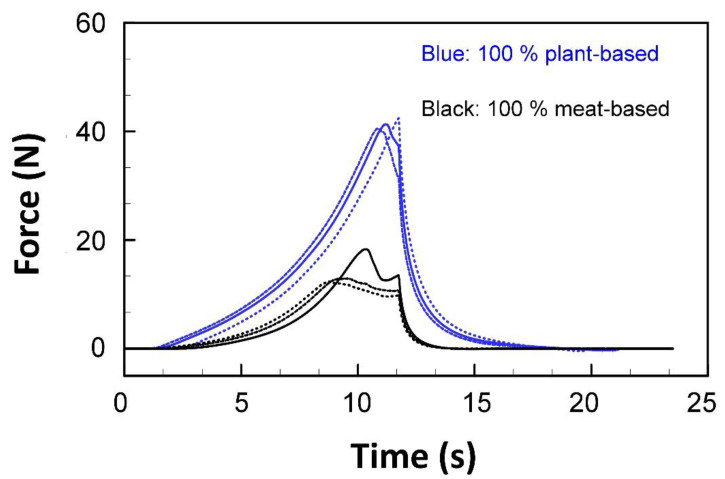
Compression response of commercially available hotdogs made with plant-based ingredients (blue) versus meat (black) at a compression speed of 1 mm s^−1^.

*Tensile and compression tests* are most commonly carried out in meat analog studies. During these tests, the force to compress or elongate a material over a defined length is recorded (Figure 3). Different test protocols and measurement geometries, as well as different fixtures, are available for different purposes. For meat analogs, the published data to date has mainly been obtained by texture-profile analysis tests, tensile tests, hardness, and cutting strength measurements [87]. During *texture profile analysis*, also known as “double compression test”, a sample of defined geometry (often cut out using a corer) is compressed and relaxed twice between parallel plates that have a larger diameter than the sample at a defined speed. The main idea behind developing this method was to mimic a twofold chewing stroke [88]. However, this test has been widely used in meat analog studies [15,87,89,90,91]. The sample is compressed between the plates to a relative deformation (often 50 to 75% of its initial height) and the force for compression (downwards) and relaxation (upwards) is measured. The results obtained by texture profile analysis reported in the literature include hardness (maximum force during the first compression peak), resilience (downstroke area under the first compression peak/upstroke area under the second compression peak), springiness (time to reach peak during the second compression/time to reach peak during the first compression), and chewiness (hardness × springiness × area under the second compression peak/area under the first compression peak) [15]. However, we want to note that there are many more parameters that can be obtained from such an analysis.

Another major goal in meat analog processing is to obtain fibrous structures that show anisotropic properties. To quantify the extent of anisotropy, *tensile stress tests* have been carried out. The meat analog is cut in defined shapes parallel and perpendicular to the shear flow (i.e., the directions of the fibers) with a known cross-sectional area that is needed to calculate the normal stress and fixed into a texture analyzer using compatible clamps. The meat analog is subsequently deformed at a constant deformation rate of typically 0.5–1 mm s^−1^ and the stress anisotropy index is calculated using Equation (1) from the obtained true stress at fracture [92,93]:

(1) $$AI=\frac{\sigma_{\mathrm{parallel}}}{\sigma_{\mathrm{perpendicular}}}(-)$$

Here, *AI* is the anisotropy index, *σ*_parallel_ (Pa) is the (true) maximum stress value in the parallel direction, and *σ*_perpendicular_ (Pa) is the (true) maximum stress resulting from perpendicular deformation. The corresponding strain anisotropy index is calculated from the maximum strain measured parallel and perpendicular to the specimen [30,94]. However, even though the anisotropy indices are useful to quantify the degree of created anisotropic structures, they should not be used as a single parameter but combined with visual observations and microscopy (see below) since it has been reported that in some cases high anisotropy indices are obtained without the presence of extensive fiber formation [64].

Lastly, *cutting force and shear tests* are performed to evaluate the “hardness” as a parameter to quantify the extent of structure and texture formation in produced meat analog samples [95]. Again, samples are cut into defined shapes and then placed below a knife-shaped geometry, which is lowered at a constant speed (typically 1–10 mm s^−1^) and the force, as well as the path to cut the specimen, is recorded. The results are reported either as maximum cutting force or the force obtained at a defined sample depth. To measure the cutting force, different geometries have been used in meat analog studies, and samples have been cut with different tools including knife geometries, Kramer, and Warner–Bratzler shear cells [14,63,96].

### 4.3. Oral Processing

Even though the oral processing behavior has not been described in meat analog studies, food scientists should consider using this approach in the future to better understand the chewing behavior and to reduce the knowledge gap between instrumental testing (dynamic shear rheology and normal force tests) and sensory trials. An advantage of using oral processing analysis is that it does not depend on the reports of panelists who may need time-intensive training, but oral processing delivers actual and quantifiable data of the events taking place during chewing [97].

**Figure 4:**
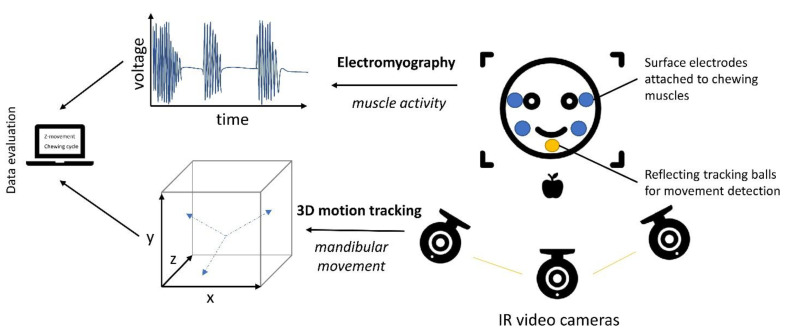
Schematic overview of oral processing analysis using electromyography and three-dimensional (3D) motion tracking using infrared video cameras.

Oral processing instruments consist of two main parts and require the participation of panelists: a jaw-tracking system and surface electrode pads to measure muscle activity using electromyography (EMG) (Figure 4). Several methods have been developed to track the movement of the mandibular during mastication. Most food scientists investigating the oral processing behavior have utilized the jaw-tracker 3D (JT-3D^TM^) system that is manufactured by BioResearch Inc. (Milwaukee, WI, USA) or using (infrared-) camera systems with or without markers [98,99,100].

Overall, the recordings will report the muscle activity of the jaw by EMG, commonly the superficial masseter and anterior temporalis from the EMG and, depending on the system, jaw-movement parameters by motion tracking such as opening velocity and movement in vertical, medial-lateral, and anterior-posterior directions are recorded [101]. The data is then used to calculate parameters like the number of chews, chewing frequency, and chewing cycle duration [98].

Oral processing would be a fruitful area for further work, helping to understand how material parameters (e.g., anisotropy index and hardness) are linked to oral properties (e.g., number of chews) and sensory characteristics (e.g., perceived firmness). This information may then help to design meat analog products that more closely mimic the oral processing behavior of meat and processed meats. For example, we could envision that oral processing studies could help to provide further insights into how the microstructure of meat and meat analogs, e.g., length of fibers and bonds between the fibers, influence chewing behavior during mastication.

### 4.4. Sensory Evaluation: Descriptive and Affective Tests

Research on meat analogs often includes the production of food-grade samples that are evaluated by sensory panels or consumers [102]. The evaluation of sensory characteristics helps to reveal the texture and flavor perception during consumption. As the aim of meat analogs is to mimic meat products as close as possible, the goal of sensory evaluation often is to determine the similarities between these products to their traditional meat product. While sensory studies are broadly classified as discrimination—(difference testing), descriptive—(perceived intensities), and affective-tests (likeness of products), studies carried out in the meat analog realm have only employed descriptive and affective testing methods, which will be described below [103].

*Descriptive tests.* During descriptive sensory tests, panelists are asked to rate given attributes (i.e., descriptors) of foods on a pre-defined scale, or in some cases also provide attributes by themselves.

Meat analogs have been characterized using different attributes including “fibrousness”, “firmness/hardness”, “juiciness”, “elasticity”, “beany”, “brittleness”, “earthy”, “chicken”, “crumbly”, “moist”, “tenderness”, and more general attributes, such as “taste”, “flavor”, and “smell” [12,29,95,104,105,106,107]. The intensities of each of these attributes are rated on a scale. A major challenge is to define the attributes and their intensities, and thus the ratings can vary across individuals. To overcome this, it is advised that descriptive sensory evaluations include between 8 and 12 panelists and that the panel undergoes training, often 20 or more hours, prior to the sensory trial to calibrate the panel with the defined attributes. For example, Savadkoohi [105] carried out an introduction session for the panel during which they compared and discussed the sensory attributes of regular sausages and processed meat analogs.

Scales are typically set in the range between 1 and 9 and are enclosed by anchor words, such as “1 = soft”, “5 = firm”, “9 = hard”. If possible, the trials should be carried out in testing booths with standardized illumination at constant sample temperature, and panelists should be provided with water to minimize carryover between products [29].

*Affective tests.* A well-established method to assess the liking of product attributes and overall acceptance of a meat analog is to use an affective test. In this method, non-trained consumers are asked to rate their liking of a food. Consequently, affective tests are a valuable method to receive fundamental feedback about the product.

Different scales have been developed over the years, but the 9-point hedonic scale ranging from “dislike extremely” to “like extremely” is still used frequently [108]. However, other scales such as the labeled affective magnitude scale and the just-about-right scales are also common in the field of food sensory science [109,110]. Especially just-about-right scales offer the advantage to combine the acceptance and intensity of specific sensory attributes at the same time (for example meaty flavor), which can help drive product development and/or quality optimization [111].

Affective tests have been used to assess the acceptance of meat analogs based on pea, wheat, peanut, chickpea, mycoprotein, and soy protein [7,90,105,112,113]. However, different scales and testing procedures have been used, which makes it difficult to compare studies. For example, Yuliarti, Kiat Kovis, and Yi [7] used a scale of 1 to 5, while a 9-point hedonic scale was used by Rehrah et al. [112]. This emphasizes the need for standardized methods also in sensory trials performed on meat analogs.

### 4.5. Practical Considerations for Texture and Sensory Analyses

In this section, we highlight some issues that should be carefully considered and accounted for when carrying out texture, mouthfeel, and sensory analysis of meat analog foods. For *texture and rheology analyses*, the sample dimensions should be kept constant, which usually means that samples should be prepared in a consistent and well-defined manner, to ensure an equal stress acting on the measurement device or the mastication muscles. Many laboratories use cutting systems, such as different types of corers or similar cutting devices, to obtain defined shapes, which is especially important for normal force measurements. Second, researchers often cover the surfaces of the measurement cells in rheometers with sandpaper to prevent slipping effects, which can cause erroneous results on meat analog samples [6]. Third, meat analog samples should not be compressed too much before carrying out rheology measurements. This is because excessive mechanical forces can lead to water loss or promote structural changes in protein or fat structures, thereby altering the subsequent response of the material to an applied shear stress. Lastly, depending on the water holding capacity meat analogs may be prone to moisture loss during storage and measurements. Samples should not be stored too long without covering before any tests, and the sample should be covered with silicon oil around the exposed surfaces during rheology measurements.

For *sensory evaluation*, analyses should be carried out following best-practicing methods, recruiting an appropriate number of panelists, and establishing appropriate controls. Products should always be presented using a controlled random sampling order, a uniform sample size, controlled for temperature, and be presented blinded with a three-digit blinding code and under controlled lightning to help reduce bias. Additionally, panelists should be offered tap water for rinsing between samples to neutralize taste receptors. In terms of panelist recruitment, best practice for descriptive testing should include 8–12 trained panelists. In contrast, affected consumer tests should include 60–120 panelists to account for the variability of the public. Finally, careful consideration should be made in determining a control (i.e., is real meat always the best choice?).

### 4.6. Practical Examples of Utilization of Texture and Sensory Analyses

In a study on the texturization properties of plant-based proteins, researchers explored how soy protein and wheat gluten blends behaved during fiber formation to understand the parameters leading to fibrous structures [114]. The authors used time-domain nuclear magnetic resonance to understand how the absorbed water is distributed among the different phases and how processing may alter the absorption of water. Additionally, a *closed cavity and small amplitude oscillatory rheology* was used to gain insights into the response of the protein structure to external stresses. An important outcome of this study was that phase separation of the different proteins occurred at high protein concentrations, which altered the water distribution among the phases, thereby affecting the overall rheological properties. This study also revealed that the tendency of soy proteins to absorb more water results in a higher volume fraction for this phase (i.e., a lower soy protein concentration in the separated soy protein phase) compared to the wheat gluten phase, which leads to an almost similar rheological response for both phases, if calculated on a volume fraction basis. This was unexpected because it was assumed that the rheological properties differ between the two proteins, since soy proteins and gluten proteins have different size and solubility characteristics. The study thus concluded that similar rheological properties of the separated phases are a prerequisite for the deformation during fibrous structure formation based on superimposed shear forces.

*Texture and sensory analysis* have been combined in a few studies [113,115]. Grahl et al. [106] fortified a meat analog with the cyanobacterium *Arthrospira platensis* (Spirulina). Soy protein-based meat analogs were produced using high moisture extrusion and the impact of spirulina content, extrusion temperature, screw speed, and moisture content on their properties was determined. The meat analogs were analyzed by a trained sensory panel that partly developed the descriptive descriptors, texture profile analysis, and by cutting force tests. The panelists assessed the meat analogs with different descriptors that included smell, color, texture, and taste attributes. For example, “brittle” was used as a descriptor for texture, whereas “umami” was used as a descriptor to assess the aftertaste. One interesting finding of this study was that the incorporation of spirulina up to 50% still resulted in the formation of a fibrous texture during extrusion at low moisture contents (57%), while the cutting force, as well as the hardness of the meat analogs, were not significantly altered by spirulina incorporation. However, the intensity of the odor, flavor, aftertaste, and color increased at higher spirulina content, which is most likely because of the intrinsic strong taste and intense color of spirulina. Additionally, the texture became less elastic, less fibrous, and had a softer structure with increasing spirulina concentration. Overall, the study concluded that it might be feasible to partially incorporate spirulina into a soy-based meat analog product.

Last, there is a relatively small body of literature on the flavor chemistry of meat analogs using analytical techniques to detect the volatile composition of extrudates. Even though we do not discuss this in great detail in this review, we want to highlight the study of Guo et al. [116], which used a GC-MS approach to measure the volatile retention of meat analogs formed by a high-moisture extrusion process and detected a great variety of flavor compounds, including esters, alkanes, alkenes, phenols, aldehydes, and alcohols. However, more studies need to be carried out to detect more volatile compounds in meat analogs with different raw materials and under different processing conditions, and to compare them with real meat products.

## 5. Particle Characteristics

Meat analogs typically contain various kinds of particulate matter, including protein aggregates, biopolymer microgels, and/or fat droplets. These particles are often used to provide desirable textural and nutritional properties to meat analogs [117,118]. Several approaches have been developed to replace animal-fats with other substances, including solid plant fats, hydrogenated vegetable oils, crystalline particles, oleogels, structured emulsions, fibers, and structured proteins [119,120,121,122]. To analyze the characteristics of the fat-replacer network formed, several methods have been implemented by food scientists, which will be reviewed here.

### 5.1. Particle Size: Microscopy and Light Scattering

The size and shape of fat replacers are important because they influence the appearance, stability, textural properties, and mouthfeel of foods [123]. Food scientists have therefore developed and employed a variety of methods to quantify the dimensions and morphology of fat replacers.

The size of the fat droplets is of special interest in fat-replacers prepared by emulsifying approaches, such as emulsion gels [54]. Such concentrated emulsion systems are prone to destabilization upon dilution and stirring, which is often necessary for droplet size analysis by light scattering techniques. For these reasons, microscopy and image analysis techniques that do not require dilution have been employed.

**Figure 5:**
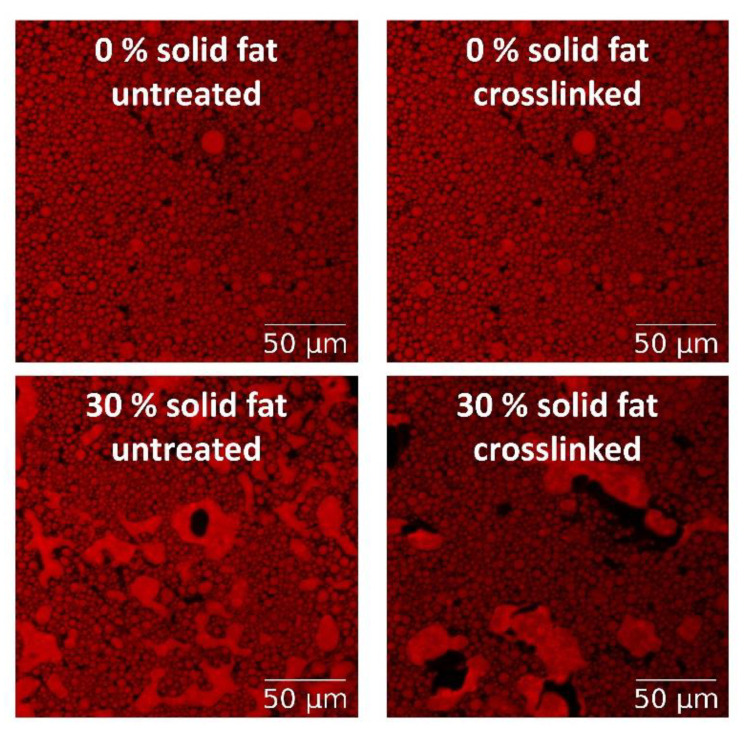
Confocal laser scanning microscope (CLSM) images of uncrosslinked and crosslinked with microbial transglutaminase emulsified fat crystal networks with a total fat content of 70% stained with Nile red.

*Confocal laser scanning microscopy (CLSM)* is widely used because it has a higher resolution than conventional light microscopy and because it is possible to carry out optical sectioning. It is particularly useful for visualizing oil droplets with dimensions around 1 μm (for a more in-depth description, see part “appearance and microstructure”). The oil phase of the emulsion is mixed with a fluorescence dye, such as Nile red, to distinguish the dispersed phase from the surrounding continuous phase (Figure 5). Subsequently, the digital image obtained is analyzed using image analysis software to determine the area of each droplet and to calculate the particle diameters [40]. The following equations are useful to calculate the individual and mean droplet diameters. First, the circular diameter of each droplet is calculated using basic geometry:

(2) $$d\;=\;2\sqrt{\frac{A}{\pi }}(m)$$

Here, *d* represents the circular diameter of the droplet, and *A* the circular area of each droplet obtained from the image analysis. Based on the droplet size distribution, the mean diameter, such as the Sauter diameter *d*_32_, is calculated employing the specific surface area:

(3) $$d_{32}=\frac{6}{S_{V}}\;(m)$$

Here, *S*_V_ the specific surface area in m^−2^ m^−3^:

(4) $$S_{V}=\frac{S_{\mathrm{total}}}{V_{\mathrm{total}}}\;(m^{-1})$$

Here, *S*_total_ in m^2^ and *V*_total_ in m^3^ are the total spherical surface area and the total spherical volume, respectively, calculated from the droplet diameter using Equation (2) assuming spherical droplets. This method has proved to be useful in studies of emulsion gels intended to be used in raw fermented and cooked salami analog foods [40,54].

Besides microscopy, *light scattering* is a powerful tool to analyze the particle size distribution of droplets and other types of particles. Even though it is sometimes difficult to measure the particle size distribution of concentrated emulsions, other types of fat replacer particles have been successfully investigated with light scattering devices. Predominantly, two different light scattering techniques have been utilized to gather information about the particle size distributions: static light scattering and dynamic light scattering [81,124].

Particle sizing instruments based on static light scattering fire a monochromatic light beam through a diluted sample and measure the resulting scattering profile (intensity vs. scattering angle). The nature of the scattering profile depends on the size of the particles in the sample: as the particle size increases, so does the intensity of the light that is scattered in the forward direction. Based on the detected scattering profile, a mathematical model (Mie theory) is used to calculate the particle size distribution. The particle size distribution is subsequently used to calculate characteristic parameters of the particles, such as mean diameters or polydispersity index. The static light scattering method has been successfully used to measure the size of various kinds of fat replacers, including microparticulated whey proteins, whey protein-pectin complexes, micronized cornstarch, and oleogels [81,125,126,127].

Particle sizing instruments based on dynamic light scattering are based on measurements of the random Brownian motion of particles in a dispersed system: the smaller the particles, the faster their movement. Nevertheless, Brownian motion also depends on temperature and solution viscosity, and so these parameters should be fixed and known. During the measurement, a monochromatic light beam is directed at the sample. The intensity of the scattered light is then measured over time, typically at an angle of either 173° or 90° depending on the instrument used. As the particles in the dispersion move, the measured scattering intensity fluctuates caused by interferences as a function of time, which is correlated to the particle size [128]. For small particles, the scattering intensity changes faster than for larger particles due to their higher diffusion coefficient. This time-dependent change in intensity is used to create a correlation function and a correlogram that is used to calculate the mean particle diameter, polydispersity index, and particle size distribution.

### 5.2. Charge: Zeta-Potential

Food grade fat replacer particles often carry an electric charge generated by ionizable side groups, such as -${\mathrm{NH}}_{3}^{+}$ or -COO^−^. Knowledge of the particle charge is important for a number of reasons: (i) creation of a stable disperse system; (ii) formation of electrostatic complexes; (iii) understanding ingredient interactions; and (iv) understanding potential mouthfeel effects. The charge of small particles is usually characterized by their ζ-potential, which is the surface potential that develops due to the electrical double layer around a charged particle at the so-called “slipping-plane”, which is generated during particle movement at a short distance away from the particle’s surface [128].

The ζ-potential is commonly measured using a laser Doppler electrophoresis instrument. These instruments are equipped with a laser that is split into two beams: a reference beam and a test beam that is used to detect the movement of the particles in the sample. The sample is loaded into a cuvette equipped with two electrodes and a voltage is applied. This causes the particles to move toward the oppositely charged electrode at a velocity that depends on their size. The particle velocity can be measured by combining laser Doppler velocimetry with phase analysis light scattering, which can then be used to calculate the electrophoretic mobility [129]. The zeta potential can then be determined from the electrophoretic mobility, provided the viscosity, dielectric constant, and ionic strength of the surrounding fluid are known:

(5) $$U_{E}=\frac{2\zeta \varepsilon_{0}\varepsilon_{r}f(\kappa a)}{3\eta }(m^{2}V^{-1}s^{-1})$$

Here, *U*_E_ is the electrophoretic mobility, *ε*_r_ is the relative dielectric constant of the surrounding solution, *ε*_0_ is the dielectric constant of a vacuum, *ζ* is the zeta potential, *η* is the apparent viscosity of the surrounding solution, and f(*κa*) is Henry’s function, which depends on the Debye screening length (*κ*) and particle radius (*a*).

### 5.3. Practical Considerations for Particle Analyses

Particle size and *ζ*-potential measurements are prone to measurement errors and misinterpretation and must therefore be carried out carefully. In this section, some of the most important factors to consider are outlined.

For *light scattering techniques*, there is a fundamental difference between dynamic and static light scattering methods and each technique has its strengths and weaknesses. Static light scattering devices report a volume-weighted distribution, which can be converted into an area- or number-weighted distribution, but this conversion may lead to errors. Static light scattering devices can detect a wide range of particles ranging from about 30 nm to 3 mm. In contrast, dynamic light scattering devices report an intensity-weighted distribution, which can be converted into a volume-, area- or number-weighted distribution, but again this may lead to errors. In addition, dynamic light scattering devices are more suited for analyzing relatively small particles (0.3 to 3000 nm) that are not prone to gravitational separation and have a low polydispersity index (i.e., monomodal dispersions). Overall, both techniques are useful to analyze fat replacer systems for meat analogs but should be chosen carefully, depending on the expected size of the particles being tested.

For both dynamic and static light scattering measurements, the samples have to be diluted to prevent multiple scattering events. Correct dilution is often critical to obtain accurate values. It is essential to use the same buffer for dilution as for the sample preparation, with the same pH and ionic strength. Buffers with different compositions may result in aggregation or dissociation of particles and thus not representing the real conditions in the sample. In general, the use of distilled water for diluting samples is not recommended as this can change the pH, charge, and aggregation state of the particles.

Finally, static light scattering requires the input of real and imaginary indices that are often not known for new raw materials. In the case of emulsion-based systems, some studies have used the refractive index of the oil phase that is often reported in the datasheet of the oil [130,131]. Moreover, some light scattering devices have built-in methods to approximate the refractive indices, but in doubt, the refractive indices should be determined using a refractometer.

The *zeta potential measurement* is also very sensitive to the method of dilution and sedimentation or creaming. As for size measurements, the same buffer as for sample preparation should be used to reflect the real charge conditions in the sample. In some cases, samples may be diluted to reduce the ionic strength to reduce the conductivity that can cause sample and cuvette deterioration. However, one needs to keep in mind that a reduction of the ionic strength may lead to a change in the zeta potential due to an increase in the Debye screening length.

### 5.4. Practical Examples of Utilization of Particle Characterization

Although food scientists have developed a variety of techniques and utilized many different ingredients to design fat replacer systems, some new promising technologies are still emerging that might be useful for meat analog formulations. Many meat analog manufacturers still use hydrogenated oils or solid plant fats as fat replacers, which may not help consumers to meet the dietary recommendations regarding saturated fat intake [11].

A challenge of some techniques to produce fat replacers for meat analogs is the lack of formation of structures that scatter light and, thus, the absence of turbidity or a strong color, and the need for high processing temperatures [132]. Animal fat particles appear whitish and a fat replacer should mimic this appearance. To mimic this appearance, an emulsion-based approach was adopted in the study of Dreher et al. [40] that showed the possibility of producing a fat replacer system with plant-based ingredients. The authors homogenized canola oil with different amounts of fully hydrogenated oil at 65 °C with soy protein at a total lipid content of 70% and subsequently cooled the emulsion down to 37 °C. To induce inter-particle crosslinking and mimic the material properties of animal fat tissue, the emulsion was crosslinked with microbial transglutaminase to form covalent bonds among the amino acid residues at and between the interfaces. The authors analyzed the particle size distribution using a microscopy approach and measured the textural properties by normal force tests and rheometry. Overall, the approach proved to be useful to tune the elastic and plastic behavior by adapting the solid fat content, but it was concluded that solid fat contents beyond 30% are not suitable as a fat replacer because of the increased plastic response of the emulsion gel. Microscopy techniques have also been used to get data about fat-replacer particles in the upper micrometer range (<300 μm). These particles have been analyzed in the food matrix using light microscopy in a fixed state or after treatment with a dye [133,134]. The particles were visualized by cutting the sample from the prepared food matrix and fixing the sample in formalin. After dehydration, the objects were soaked in xylene and in paraffin to prepare the samples for sectioning. Thin sections with a thickness below 10 μm were subsequently prepared with a microtome and transferred onto glass slides, which were dyed with Schiff’s reagent and hematoxylin before visualizing their microstructure with a light microscope [133,134,135].

An oleogel approach was taken in the study of Patel et al. [126]. Initially, the authors prepared an O/W-emulsion with methylcellulose and xanthan gum. The emulsion was subsequently dried in an oven at 50 to 80 °C, producing an oleogel with >97% oil content. The study employed static light scattering for particle size measurement and rheometry to reveal the structural properties of the oleogels. The authors stated that the process yielded a stable emulsion without coalescence of oil droplets during the concentration through drying. Additionally, the oleogels exhibited a high gel strength and a shear-thinning flow behavior. While this study did not demonstrate the use of the fat substitute in meat analogs, it did demonstrate the use in bakery products and the results could potentially be transferred to analog products.

Lastly, dynamic light scattering has been employed to measure the particle sizes of protein-carbohydrate complexes that potentially could be used as fat replacers, such as β-lactoglobulin-carrageenan and β-lactoglobulin-pectin complexes [136]. In another study, the size of untreated and hydrolyzed oat β-glucan was measured by dynamic light scattering to determine the degree of aggregation [136]. A number of researchers have used the same device to carry out electrophoresis measurements to determine the charge of ingredients used to develop meat analogs. For instance, the *ζ*-potential values of different fat replacer particles have been measured, including cornstarch nanocrystals, whey protein-pectin complexes, chia protein-gum complexes, and microemulsions [137,138,139,140]. Further studies may assess the influence of electrostatic interactions on the matrix binding of fat substitutes within meat analogs, which is of special interest for emulsion-based fat replacer systems.

## 6. Appearance and Microstructure

In this section, techniques available to analyze the appearance and microstructure of plant-based meats are briefly considered. The visual appearance of foods is an important aspect of the sensorial perception and acceptance of a food product [141]. Many researchers show photographs of the meat analogs they have developed to provide information about their structure and visual appearance [14,17,30,63,115]. However, more sophisticated methods have also been utilized to provide more detailed information about the microstructural properties and to quantify the visual parameters.

### 6.1. Color: L*a*b*

One characteristic of many real meat products is their red (e.g., salami, or whole meat cuts) or white to beige color (e.g., Frankfurters or Bologna sausage), which is caused by myoglobin, its derivatives, and the denaturation of muscle proteins. To fully mimic meat, different food colorants are typically added to meat analog products on the market, such as beet juice extract, soy leghemoglobin, carrot juice extract, and lycopene [11]. In academic studies, the majority of work has not focused on the effects of specific colorants on the color of meat analogs, but instead, has used color as a parameter to understand and compare how the structuring and cooking processes affect the color by chemical reactions, such as the Maillard reaction and the degradation of residual pigments [7,87,95,96,113].

Typically, the surface color is measured with an instrument colorimeter and is reported using the CIELAB color space with D65 as a standard illuminant. Colorimeters are usually equipped with a pulsed xenon arc lamp, which illuminates the sample with a standardized light beam. The light reflected from the sample surface is then collected using photocells and is used to calculate the coordinates in the color space [142]. Before the measurement, the colorimeter is calibrated with a white plate to standardize the reported results. The results are reported in the CIELAB color space, where L* describes the lightness of the sample (0 = black, 100 = white), whereas a* ranges from green (−) to red (+), and b* ranges from blue (−) to yellow (+). Moreover, the color difference Δ*E* Equation (6) has been reported for meat analog studies. A Δ*E* greater than 2 is often considered to be a recognizable color difference [143]:

(6) $$\Delta E=\sqrt{{\left(L-L_{0}\right)}^{2}+{\left(a-a_{0}\right)}^{2}+{\left(b-b_{0}\right)}^{2}}$$

Here, *L*, *a*, *b* are the values of the sample, and *L*_0_, *a*_0_, and *b*_0_ are the initial color values (e.g., before processing).

### 6.2. Image Processing Method to Analyze Fibrousness

In addition to tensile strength tests (see Section 4.2), image analysis has been proven useful to calculate a fiber index value. Ranasinghesagara, Hsieh, and Yao [144] demonstrated that an image processing approach based on preprocessing, edge detection, Hough transform, and region of interest (ROI) analysis can reliably report the fibrousness of a soy meat protein analog. Images were taken from a dissected meat analog and were preprocessed to standardize the pixel intensity and remove excessive background. Edges were subsequently detected using a Sobel gradient edge detector and the images were transformed into grayscale. The image was then used to carry out a Hough transformation, which transforms all pixels in a line segment into a corresponding single point in a parametric space [144]. The Hough transformation basically detects geometric shapes using an algorithm that transforms pixels that are on the same line into one point in the parametric Hough space. The brighter the point in the Hough space (i.e., the higher the pixel value), the longer the actual line because it consists of more pixels in the same geometrical space. As the last step, the intensity of the pixels in the Hough space is evaluated and compared in defined regions in the parametric space. The more evenly the pixel intensity along the different regions is scattered, the more uniform the produced fibers, and vice versa.

### 6.3. Microstructure: CLSM and SEM

A major aim during the production of meat analogs is the transformation of globular protein structures into a fibrous matrix. To evaluate the structure-forming properties of the employed processes and raw materials, scanning electron microscopy (SEM), confocal laser scanning microscopy (CLSM), and optical microscopy have been employed [7,14,26,29,30,64,87,95]. To achieve a higher resolution compared to light microscopes, electron microscopes use an electron beam that has a much shorter wavelength in the lower picometer range. 

Typically, an *SEM* consists of an electron source that produces a beam of primary electrons. The beam is focused and demagnified with a column of electromagnetic lenses, and then coils are used to direct the beam across the specimen surface. To prevent any interaction with air molecules, vacuum pumps generate a vacuum in the lower Pascal range. Finally, the secondary or backscattered electrons from the surface of the sample are collected by detectors [145]. SEM has often been used to visualize the fibrous microstructure of meat analog samples, which is in a size range where other microscopy techniques often fail.

*CLSM* is also commonly used to provide information about the microstructure of meat analogs [30,54]. CLSM offers the advantage of a higher resolution compared to light microscopy, while sample preparation is still relatively straightforward. The higher resolution of CLSM is achieved by having only one conjugate focal plane for both illumination and detection, and not using wide-field illumination as in conventional light microscopy. The CLSM is equipped with a laser that produces a point illumination with a pinhole and the light emitted from the sample is detected after passing through a second pinhole that has the same focal plane as the laser before entering the detector. This set-up enables a maximum lateral resolution of 190 nm and an axial resolution of more than 500 nm [145]. Another advantage of CLSM is the possibility to acquire a 3D dataset of the specimen and thus creating 3D images of the sample. Fluorescence dyes are used to visualize targeted structures, which are in meat analogs typically fat and proteins that can be dyed with Nile red and rhodamine B, respectively [30,54].

### 6.4. Practical Considerations for Appearance and Microstructure Analyses

Like other methods, color measurements and microstructure visualization must be carried out carefully to obtain reliable and accurate results. First, L*a*b* results should always be accompanied by digital photographs of a sample to get a real impression of its overall appearance. The L*a*b* reports may show changes for the given experimental parameters, but the color information provided by the data is sometimes hard to interpret by the reader. Thus, pictures help to evaluate the appearance of the meat analog. Second, before taking SEM pictures most samples need to be pretreated by fixation, drying and coated to enhance the conductivity of the sample. These treatments may alter the microstructure of the sample and can potentially lead to results not reflecting the real conditions in the sample. To overcome this problem, environmental scanning electron microscopy (ESEM) can be used, in which samples are observed in their native state without drying and coating. However, ESEM has not been employed in meat analog studies so far and the feasibility still needs to be validated.

### 6.5. Practical Examples of Utilization of Appearance and Microstructure Analyses

In a study lead by Krintiras et al. [64], SEM images and digital photographs were used to characterize the structuring properties of a soy protein-wheat gluten blend prepared by Couette shear cell. The authors used SEM to visualize the raw material properties of the soy protein raw material, but also to show how different processing conditions affect the formation of fibrous structures, with and without rotation in the shear cell. In another study carried out by [105], the effect of the incorporation of bleached tomato pomace on the color of a meat-free sausage was studied. The experiments revealed that the meat-free sausages containing 7% (*w/w*) tomato pomace exhibited an increase in lightness and yellowness, indicating a transition of the product color to more yellow with the addition of tomato pomace.

## 7. Water-Holding and Cooking Resilience

A standard test carried out in classical meat science is the water holding capacity (WHC) and cooking loss test. Both of these parameters describe the ability of meat to retain and bind fluids within its semi-solid matrix. WHC and cooking loss tests have also been transferred to meat analog research because water holding capacity and cooking properties greatly vary between the different plant protein sources, and are affected by processing [15,105,113,115,146]. However, we have to note that different conditions have been used across meat analog studies, which makes comparison difficult. Studies should be carried out at defined centrifugation and heating conditions to ensure comparability.

The WHC is often analyzed by a centrifugal method. A small amount of sample (1 to 15 g) is weighed into a centrifuge tube and centrifuged at a moderate gravitational force (<10,000 g). After centrifugation, the water is drained and the sample weight (with removed surface water) is measured and related to the initial sample weight. To prevent reabsorption of water into the sample matrix after centrifugation, perforated centrifuge tubes can be used. The WHC is then given by:


WHC = 100 × (*m*_B_ − *m*_A_)/*m*_B_ (7)

Here *m*_B_ and *m*_A_ are the sample weights before and after centrifugation.

The cooking loss (CL) is determined using a similar weighing approach but the samples are heated to a defined core temperature for a certain time in a closed container. After heating and cooling down, the released liquid is drained and the sample weight after cooking (*m*_A_) is measured and related to the sample weight before cooking (*m*_B_):

CL = 100 × (*m*_B_ − *m*_A_)/*m*_B_ (8)

## 8. Cookability

An important quality attribute of real meat products is their versatility—they can be prepared using a wide variety of cooking methods, including broiling, boiling, frying, baking, and microwaving. For this reason, the impact of different cooking methods on the structural and physicochemical properties of selected plant-based meat analogs should be systematically tested. Meat analogs should be cut into well-defined shapes, such as cubes (1 cm × 1 cm × 1 cm), and then cooked under standardized conditions. Their structural and physicochemical properties can then be measured before and after cooking, and then compared to those of real meat samples they are designed to replace. Some recommended conditions are highlighted below:

- Microwaving: samples should be placed on a microwavable plate and then heated for different times (30, 60, 90, 120, or 240 s) at a fixed power (800 watts).
- Boiling: samples should be placed in boiling water for different times (10, 20, 30, and 40 min) under constant heating conditions.
- Baking: samples should be placed on a baking tray in a convection oven for different times (10, 20, 30, 40, 50, and 60 min) operated at a fixed temperature (350 F; 177 °C).
- Broiling: samples should be placed on a baking tray in an oven operated in broiling mode at a fixed distance below the heating element. The samples should be held for different times (5, 10, 15, and 20 min).
- Frying: samples should be placed in a heated frying pan containing a fixed amount of hot corn oil, and then held for different times (2, 5, 10, and 20 min).

After cooking, the microstructure, appearance, morphology, texture, and cooking loss of the meat analog samples should be measured. If required, the precise settings used in the different cooking procedures should be adjusted to more closely resemble those typically encountered for the particular meat analog being developed.

## 9. Proposed Standardized Sequence of Analyses

In the last part of the review, we bring together the most important methods and propose a sequence that may help food scientists and developers to standardize their methodical approach when studying and developing meat analogs. The sequence is structured along with the elemental steps of food production: raw material→process→product→consumer:

1. Raw material: after acquiring the raw materials, the proximate composition should be analyzed: moisture content by oven drying; protein content by the Dumas combustion or Kjeldahl method; fat content by Soxhlet extraction; total mineral content by ashing; and, carbohydrate content as the remainder.
2. Process: in addition to measuring process parameters like torque, revolutions per minute (rpm), temperature, pressure, and others, in-line near-infrared (NIR) spectroscopy could facilitate direct feedback about the composition of the material before, during, and after meat analog processing. Large and small deformation rheology measurements will help to understand the behavior of the material during processing. Utilizing particle size analysis by microscopy will describe the size distribution of particle-based fat replacers, which is an important criterion for its stability and mouthfeel.
3. Product: the appearance should be reported by photographic images to provide a visual report of the meat analog, as well as using instrumental colorimetry (L*a*b*). The textural properties should be analyzed at least by perpendicular and parallel normal force tests. An analysis of the water holding capacity will provide feedback about a key quality characteristic of a meat analog food product. The proximate composition tests as described above can be carried out additionally for the final product.
4. Product sensory: descriptive (trained panel) and affective (consumers) sensory trials will provide insights into the various sensorial characteristics of the meat analog and will guide scientists and developers to improve the consumption experience of the consumer.

## 10. Conclusions

With the increase in meat analog and processed meat analog sales, more and more research and development is underway to improve existing products and processes, as well as to develop new ones. To equip the researchers and developers with a set of standardized methods for this quest, we have described the most important methods already used in meat analog studies and techniques that should be employed in future studies. We believe that the outlined methods will help to accurately describe observations, effects, and plan experiments that will guide the transition to a more sustainable food system.

## Data Availability

Data sharing not applicable.
